# Antibacterial Activity of Zinc-Doped Hydroxyapatite and Vancomycin-Loaded Gelatin Nanoparticles against Intracellular *Staphylococcus aureus* in Human THP-1 Derived Macrophages

**DOI:** 10.1021/acsanm.4c03941

**Published:** 2024-09-11

**Authors:** Lizzy A.B. Cuypers, Leonie de Boer, Rong Wang, X. Frank Walboomers, Fang Yang, Sebastian A.J. Zaat, Sander C.G. Leeuwenburgh

**Affiliations:** † Department of Dentistry-Regenerative Biomaterials, Research Institute Medical Innovations, Radboud University Medical Center, Philips van Leydenlaan 25, 6525 EX Nijmegen, The Netherlands; ‡ Department of Medical Microbiology and Infection Prevention, Amsterdam Institute for Immunology and Infectious Diseases, Amsterdam University Medical Center, University of Amsterdam, Meibergdreef 9, 1105 AZ Amsterdam, The Netherlands

**Keywords:** nanoparticles, drug delivery, antibiotic, intracellular pathogens, vancomycin, zinc

## Abstract

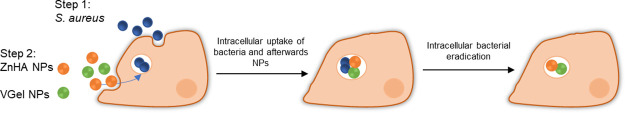

Treating bone infections with common antibiotics is challenging, since pathogens like *Staphylococcus aureus* can reside inside macrophages. To target these intracellular bacteria, we have proposed nanoparticles (NPs) as drug carriers. This study aims to investigate the efficacy of hydroxyapatite and gelatin NPs, selected in view of their bone mimicry and potential for targeted delivery, as carriers for the antibacterial agents zinc and vancomycin. Therefore, two distinct NPs are fabricated: zinc-doped hydroxyapatite (ZnHA) and vancomycin-loaded gelatin (VGel) NPs. The NPs are characterized based on morphology, size, chemical composition, cellular internalization, and intracellular bactericidal efficacy. Specifically, the intracellular bactericidal efficacy is tested using a validated coculture model of human THP-1 derived macrophages and phagocytosed *S. aureus* bacteria. Scanning electron microscopy (SEM) and Fourier transform-infrared spectroscopy (FTIR) results show that the spherical NPs are synthesized successfully. These NPs are internalized by THP-1 cells and show >75% colocalization with lysosomes without compromising the viability of the THP-1 cells. Both ZnHA and VGel NPs substantially reduce the intracellular survival of *S. aureus* compared to the direct addition of dissolved zinc and vancomycin. Concluding, our NPs are highly effective drug delivery vehicles to kill intracellular *S. aureus*, which stress the potential of these NPs for future clinical translation.

## 1 Introduction

Bone infection often leads to inflammatory destruction and ultimately necrosis of bone tissue, thus posing a significant challenge for healthcare. Such infections predominantly arise following trauma or surgical intervention, including the application of joint prostheses or orthopedic fixation devices.^1^ One of the most frequent causative pathogens of bone infections is *Staphylococcus aureus*. This pathogen is an opportunistic Gram-positive bacterial species commonly residing in the skin and nasal cavity, which has the capacity to cause severe and life-threatening infections upon invasion of the bloodstream or bone tissue. Although *S. aureus* is traditionally recognized as an extracellular pathogen, increasing evidence highlights its ability to persist intracellularly inside mammalian cells like macrophages.^2,3^ This intracellular survival creates a formidable challenge for conventional treatment strategies based on systemic administration of antibiotics, not only for bone infections but also for many other types of infections in nonosseous tissues caused by intracellular pathogens. Antibiotics such as vancomycin, which are commonly used to treat bone infections, are unfortunately poorly able to cross lipid cell and organelle membranes.^4,5^ Furthermore, once internalized, *S. aureus* enters a “persister” state characterized by reduced metabolic activity. Such a state renders *S. aureus* highly resistant to antibiotics even at high concentrations,^6^ which may even induce recurrent and chronic bone infections at later stages.^2^ Hence, there is an urgent need to develop novel antibacterial treatments that can effectively target and eliminate intracellular *S. aureus*.

To this end, it is crucial to develop effective antibacterial drug delivery carriers that can cross the cell membrane. Nanoparticles (NPs) have emerged as promising candidates to this end, owing to their ability to penetrate tissues and undergo cellular internalization.^7,8^ The use of NPs for delivery of antibiotics has extensively been described in the literature.^9−11^ However, the use of antibiotics is inevitably associated with the risk of promoting antibiotic resistance. To reduce antibiotic resistance, there is growing interest in exploring alternative or adjuvant strategies compared to traditional antibiotics. For this purpose, metal ions like zinc,^12^ silver,^13^ and copper^14^ have been identified as promising candidates with the capacity to exert antibacterial effects. These ions can also be used in combination with antibiotics to exert dual effects against bacteria.^15^

In the literature, multiple carrier systems for the delivery of either antibiotics or ions have been proposed, including silicon-based NPs,^16^ chitosan NPs,^17^ and metal-based NPs like gold^18^ and silver,^19^ with recent developments highlighted in a review by Wang et al.^5^ However, effective treatment of intracellular bone infections requires not only effective delivery of therapeutic agents but also enhancement of bone formation to counteract bone loss due to infection. Consequently, NPs that mimic the composition of bone tissue are particularly attractive from this perspective. Bone primarily consists of type I collagen and hydroxyapatite.^20^ Consequently, gelatin, a derivative of collagen, and hydroxyapatite, corresponding to the mineral phase of bone, are evident material candidates for this purpose. Gelatin (Gel) NPs are renowned for their biocompatibility and tunable biodegradability.^21^ Furthermore, Gel NPs are well-established as effective carriers for antibiotics.^22−24^ Similarly, hydroxyapatite (HA) NPs have emerged as promising vehicles for ion delivery, since the apatite structure of HA can store various antibacterial metal ions like zinc,^25^ silver,^26^ and copper.^26,27^ Spherical HA NPs have been frequently recognized to more effectively stimulate bone regeneration as compared to other shapes like rod-, needle-, or plate-like NPs.^28^

Despite these advancements, the intracellular bactericidal efficacy of spherical HA and Gel NPs as drug carriers for ions and antibiotics remains unknown. Therefore, this study aims to explore the intracellular bactericidal effectiveness of these two types of NPs as carriers for metal ions and antibiotics. Specifically, zinc and vancomycin were selected to represent these two categories of agents. Accordingly, we developed two unique types of NPs: (i) zinc-doped hydroxyapatite nanoparticles (ZnHA NP) and (ii) vancomycin-loaded gelatin nanoparticles (VGel NP). Subsequently, the cytocompatibility of these NPs with human THP-1 derived macrophages was systematically assessed. THP-1 cells were selected since these cells exhibit several relevant characteristics of human macrophages and comprise a homogeneous and reproducible cell population.^4^ To investigate the efficacy of local delivery of zinc and vancomycin against intracellular bacteria, a previously reported coculture model was adapted and validated, wherein THP-1 cells are infected by means of phagocytosis of *S. aureus*.^29−31^ Finally, the efficacy of the NPs against intracellular *S. aureus* was evaluated using the above-described coculture model.

## 2 Experimental Section

### 2.1 Synthesis of Zinc-Doped Hydroxyapatite Nanoparticles

**Figure 1:**
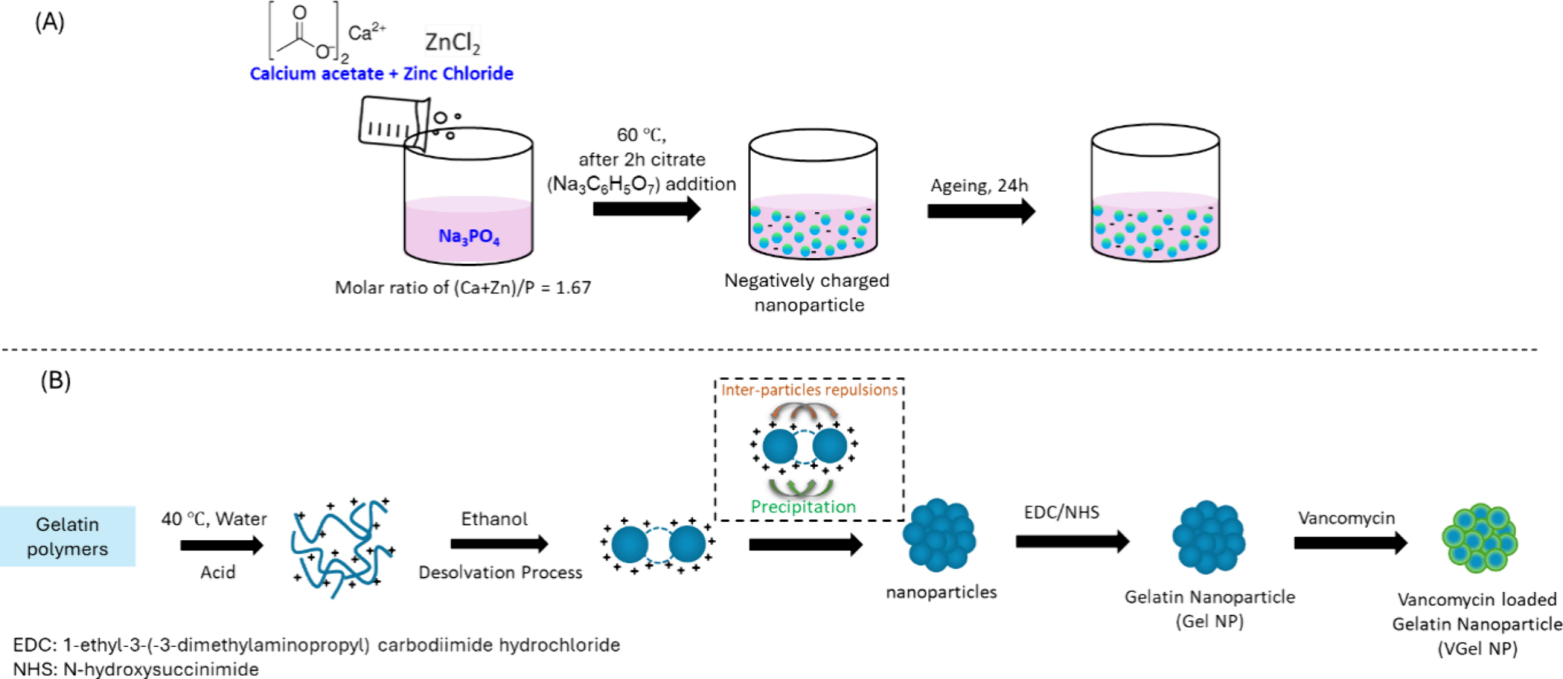
Schematic representation of the synthesis and fabrication of (A) zinc-doped hydroxyapatite NPs and (B) vancomycin-loaded gelatin NPs.

ZnHA NPs with a molar [Ca+Zn]/[P] ratio of 1.67 were synthesized according to a recently published method by Cuypers et al.^25^ (Figure 1A). In brief, a 50 mM trisodium phosphate solution (Na_3_PO_4_·12 H_2_O; Sigma-Aldrich, Saint Louis, MI, USA) was heated to 60 °C. Appropriate amounts of 100 mM zinc nitrate (Sigma-Aldrich) and 83.5 mM calcium acetate monohydrate (Ca(C_2_H_3_O_2_)_2_ H_2_O; Sigma-Aldrich) solutions were added to reach zinc molar percentages of 0, 10, 15, and 20 mol %. Subsequently, tribasic sodium citrate (Na_3_C_6_H_5_O_7_; Merck, Darmstadt, Germany) at a concentration of 9.4 mg/mL was added for stabilization of the NPs. The solution was stirred continuously for 24 h at 1000 rpm at 60 °C, and the resulting precipitate was thereafter centrifuged and washed three times with demineralized water. Finally, the NPs were either stored in suspension at 4 °C for up to 3 days or lyophilized for 48 h and stored at room temperature.

### 2.2 Synthesis of Gelatin Nanoparticles and Diffusional Postloading of Vancomycin

Gel NPs were prepared by a desolvation process using ethanol (Figure 1B). Briefly, 1.25 g of gelatin type A or type B (Gel A or Gel B, from porcine skin, 300 Bloom (type A) and 247 Bloom (type B), isoelectric point (IEP) ∼9 and ∼5, Rousselot, Gent, Belgium) was dissolved in 25 mL of demineralized water under constant stirring at 500 rpm at 40 °C. The pH was set to 3.0 using 1 M HCl (37% fuming, Merck), and 100 mL of ethanol was added dropwise (5 mL/min) under stirring at 1000 rpm to induce first gelatin desolvation and then aggregation into Gel NPs. Thereafter, the Gel NPs were cross-linked by 5 mM, 1-ethyl-3-(3-dimethylaminopropyl)carbodiimide (EDC, Sigma-Aldrich) and 1 mM *N*-hydroxysuccinimide (NHS, Sigma-Aldrich). After 16 h of cross-linking, a crossflow setup based on a Sartorius Stedim Sartocon Slice filter holder equipped with a 300 kDa cutoff membrane was used to remove ethanol and wash Gel NPs with demineralized water.

In order to fabricate vancomycin-loaded Gel NPs (VGel NPs), 100 μg vancomycin (Sigma-Aldrich) was dissolved in 1 mL of a freshly prepared suspension of Gel NPs in demineralized water (8 mg/mL) and equilibrated at 4 °C for at least 12 h to allow for absorption and diffusion of vancomycin to the Gel NPs based on interactions as described previously.^32^ Afterward, the VGel NPs were centrifuged at 10.000 rpm for 10 min to remove all the unbound vancomycin, and the NPs were resuspended in phosphate buffered saline (PBS). The NPs were either stored in suspension at 4 °C for up to 3 days to minimize antibacterial agent release or lyophilized for 48 h and stored at room temperature.

### 2.3 Physiochemical Characterization of Nanoparticles

The size and morphology of the synthesized NPs were characterized using scanning electron microscopy (SEM; Sigma 300 field emission SEM, Zeiss, Oberkochen, Germany). For HA, 10 mol % ZnHA, 15 mol % ZnHA, and 20 mol % ZnHA NPs, a 1:1000 diluted suspension of NPs in demineralized water was dropped on a copper grid (10 mm diameter, 75 mesh, TED Pella, INC.) and allowed to evaporate. For GelA, GelB, VGelA, and VGelB NPs, the NPs were first lyophilized before being spread as a thin coat on the carbon tape. All SEM samples were coated with a layer of electroconductive chromium (∼10 nm) before imaging.

The hydrodynamic size of the synthesized NPs was measured using dynamic light scattering (DLS, Zetasizer, Malvern PANalytical, Malvern, United Kingdom) at 25 °C. The zeta potential of the synthesized NPs was measured in HEPES buffer (pH 7) using a Zetasizer (Malvern PANalytical, Malvern, United Kingdom).

Chemical bonds and structures in the synthesized NPs were characterized by Fourier transform-infrared spectroscopy (FTIR; Spectrum Two FT-IR Spectrometer, PerkinElmer, Waltham, MA, USA). The spectrum was recorded in the range of 400–4000 cm^–1^ with a resolution of 4 cm^–1^.

In order to calculate the amount of vancomycin bound to VGelA and VGelB NPs, 1 mg NPs were degraded with 1 mL of 2.5 mg/mL collagenase (Sigma-Aldrich) at 37 °C for 2 h. Afterward, the solution was filtered and measured by high performance liquid chromatography (HPLC; Hitachi 5160 pomp, Hitachi 5430 DAD detector, and Hitachi 5260 autosampler) at a wavelength of 230 nm.

### 2.4 Internalization of Nanoparticles by Human THP-1 Derived Macrophages

#### 2.4.1 Cell Culture

For cell culture experiments, human THP-1 monocytes (American Type Culture Collection, Manassas, WV, USA) were maintained in Roswell Park Memorial Institute (RPMI) 1640 medium supplemented with 2 mM glutamine (GIBCO, New York, NY, USA) and 10% fetal bovine serum (FBS, Sigma-Aldrich). The THP-1 cells were subcultured once a week and incubated at 37 °C in a humidified atmosphere containing 5% CO_2_. The THP-1 cells were passaged by centrifugation when reaching 10^6^ cells/mL. In the following experiments, cell seeding was performed with media enriched with 50 ng/mL phorbol 12-myristate 13-acetate (PMA; Sigma-Aldrich) to allow for adherence of cells over 48 h to differentiate the THP-1 monocytes to THP-1 derived macrophages.

#### 2.4.2 Cytocompatibility of Nanoparticles

To assess the cytocompatibility of the synthesized NPs and antibacterial agents against THP-1 cells, a cell counting kit assay (CCK8; 150 mM NaCl, 5 mM WST-8 and 0.2 mM 1-methoxy PMS in demineralized water) was performed. THP-1 cells were cultured as described above and seeded in a 96-well plate at a concentration of 100,000 cells/cm^2^. After 48 h of incubation, the culture media were replaced with 200 μL of RPMI culture media containing specific concentrations of NPs which will also be used for the intracellular killing experiments: 12.5, 25, or 50 μg/mL for synthesized NPs, or specific concentrations of vancomycin (5.5 μM) or zinc (300 μM). These concentrations were based on previous work within our group focusing on NP cytotoxicity and internalization, which indicated that concentrations above 50 μg/mL show aggregation of NP within the cells (data not shown). Moreover, the concentrations of vancomycin and zinc were selected based on their prior use in positive control groups, where they demonstrated effective killing of planktonic bacteria. THP-1 cells cultured in RPMI media without treatment served as a negative control. After 24 h of culture, the media were replaced by fresh media supplemented with 10% CCK8 reagent and incubated for an additional 2 h. The supernatants of each well were extracted, and the absorbance was measured at 450–490 nm with a spectrophotometer (BioTek, Winooski, VT, USA).

#### 2.4.3 Fluorescent Labeling of Synthesized Nanoparticles

To allow for the visualization of cellular HA NPs and ZnHA NP uptake, HA NPs and ZnHA NPs were fluorescently labeled with 5-FAM-ZOL dye (Biovinc, Pasadena, CA, USA), and 100 μL of 0.01 nM FAM-ZOL dye was added to 1 mg/mL HA NPs or ZnHA NPs suspended in PBS at pH 7.4.

To fluorescently label GelA and GelB NPs, 50 mL of 0.1 mg/mL fluorescein 5(6)-isothiocyanate (FITC; Sigma-Aldrich) dissolved in demineralized water was added to the GelA or GelB NP suspension (10 mg/mL, 200 mL). After an incubation period of 6 h at room temperature, the remaining free FITC was removed via crossflow filtration. For the visualization of internalization of VGelA NPs or VGelB NPs, 100 μg of FITC-vancomycin (Sigma-Aldrich) was added in 1 mL suspension of VGelA NPs or VGelB NPs (8 mg/mL) in demineralized water to obtain FITC-VGelA NPs and FITC-VGelB NPs. All labeled NPs were stored in a suspension at 4 °C and used without further modification.

#### 2.4.4 Internalization of Nanoparticles

THP-1 cells were cultured as described above and seeded at a density of 200,000 cells/cm^2^ in an 8-well μ-slide (ibidi, Gräfelfing, Germany) for 48 h. Labeled NPs were diluted in the RPMI cell culture medium to reach a final concentration of 50 μg/mL. Afterward, 200 μL of particle suspension was added to the THP-1 cells. As a control group, 5 μL of dissolved FITC-vancomycin (100 μg/mL) was added, while THP-1 cells were cultured with RPMI media without NPs serving as a negative control. The next day, cells were washed twice with PBS and stained with 1 μM CellTrace yellow (Invitrogen) in PBS according to the manufacturer’s instructions. Additionally, lysosomal compartments were stained with 50 nM LysoTracker deep red (Thermo Fisher, Waltham, MA, USA) in phenol-red-free RPMI medium supplemented with 10% FBS and 20 mM HEPES 30 min prior to imaging. The internalization of the labeled NPs and FITC-vancomycin was visualized using a Leica TCS SP8 SMD confocal microscope (Leica Microsystems, Wetzlar, Germany), equipped with an HCX PL APO 63×/0.40 water immersion objective and a temperature-controlled stage at 36.5 °C. The image analysis software Fiji (NIH, Boston, MA, USA) was used for the reconstruction of images, and the plugin “co-localization finder” was used to quantify the fluorescence overlay of different channels. The overlaying signal was visualized in white.

### 2.5 Coculture Model of THP-1 Cells Containing Phagocytosed *S. aureus*

#### 2.5.1 In Vitro Phagocytosis of *S. aureus*-mCherry

*S. aureus* strain RN4220 expressing mCherry fluorescent protein (*S. aureus*-mCherry) was used for in vitro studies. Bacterial inoculums were prepared by culturing *S. aureus*-mCherry in tryptic soy broth (TSB; BD Difco, Leeuwarden, The Netherlands) supplemented with 10 μg/mL chloramphenicol (Merck) shaking at 120 rpm and 37 °C to the mid logarithmic growth phase. *S. aureus*-mCherry bacteria were pelleted by centrifugation (3000 rcf, 10 min), resuspended in 1 mL NaCl and 0.25 mL human serum (H1 serum, Bio Whittaker, MD, USA), and incubated for 20 min. The inoculum was adjusted to 10^8^ colony forming units (CFU)/mL with RPMI based on the optical density value at wavelength 620 (OD_620_). The THP-1 cells were seeded as described above in 96-well plates at a concentration of 100,000 cells/cm^2^. After 48 h, the media were replaced by 49 μL of RPMI media +1 μL of the bacterial inoculum (resulting in a bacteria to cell ratio of 1:1) and incubated for 45 min at 37 °C and 5% CO_2_. Afterward, the RPMI media with bacteria were removed, and the THP-1 cells were washed three times with 50 μL of PBS and one time with 200 μL of PBS. RPMI media supplemented with 5 μg/mL gentamycin (Sigma-Aldrich) were added to control the extracellular growth of *S. aureus*-mCherry.

#### 2.5.2 Validation of the Coculture Model

THP-1 cells were seeded at a concentration of 100,000 cells/cm^2^ in an 8-well μ-slide and were allowed to phagocytose *S. aureus*-mCherry as described above. After 24 h, the THP-1 cells were washed twice with PBS, and lysosomal compartments were stained with 50 nM LysoTracker deep red as described above. The internalization of *S. aureus*-mCherry was visualized using a Leica SP8-X DLS Lightsheet microscope (Leica). The image analysis software Fiji was used for the reconstruction of images, and the plugin “co-localization finder” was used to quantify the fluorescence overlay of different channels. The overlaying signal is also visualized in white.

Additionally, a LIVE/DEAD cell viability assay (Invitrogen, Waltham, MA, USA) was used according to the manufacturer’s instructions to visualize the cells after 24 h of phagocytosis. In brief, 100 μL of 4 μM ethidium homodimer 1 and 2 μM calcein AM solution in PBS was added to the THP-1 cells and incubated for 30 min before visualization using a Leica SP8-X DLS Lightsheet microscope (Leica). THP-1 cells without phagocytosed *S. aureus*-mCherry served as the negative control.

#### 2.5.3 Visualization of Intracellular *S. aureus*-mCherry and Nanoparticles

THP-1 cells were cultured at a concentration of 100,000 cells/cm^2^ in an 8-well μ-slide and were allowed to phagocytose *S. aureus*-mCherry as described above. After phagocytosis, RPMI supplemented with 5 μg/mL gentamycin was enriched with 50 μg/mL fluorescently labeled NPs. The cells were incubated for 24 h, whereafter lysosomal compartments were stained with 50 nM LysoTracker deep red as described above. The *S. aureus*-mCherry, lysosomes, and labeled NPs were visualized by confocal laser scanning microscopy. The image analysis software Fiji was used for the reconstruction of images, and the plugin “co-localization finder” was used to quantify the fluorescence overlay of different channels. The overlaying signal is also visualized in white.

#### 2.5.4 Nanoparticle Addition to Coculture Model

THP-1 cells were seeded at a concentration of 100,000 cells/cm^2^ in 96-well plates and were allowed to phagocytose *S. aureus*-mCherry as described above. After phagocytosis, RPMI supplemented with 5 μg/mL gentamycin was enriched with 50 μg/mL NPs, and as a control, zinc (300 μM) and vancomycin (5.5 μM) were added. The cells were incubated for 24 h and afterward lysed with 100 μL 0.025% TritonX-100. The supernatant was serially diluted and spot-plated on agar plates. The agar plates were incubated for 24 h in 37 °C and 5% CO_2_ before counting the CFU.

### 2.6 Statistical Analysis

All data were presented as mean ± standard deviation (SD) of samples in triplicate (*n* = 3), unless mentioned otherwise. Statistical differences were evaluated using Prism (GraphPad software, San Diego, CA, USA). Statistical comparison of data from different compositions was carried out using one-way analysis of variance (ANOVA) with an additional multiple comparison test (Tukey) for differences between groups.

## 3 Results and Discussion

### 3.1 Physicochemical Characterization of Nanoparticles

HA and ZnHA NPs with three different zinc contents (10, 15, and 20 mol %) were synthesized using a wet chemical precipitation method as previously described.^25^ Gel NPs were produced via a desolvation method from two types of gelatin, gelatin type A (GelA) and gelatin type B (GelB), and subsequently loaded with vancomycin by simple diffusional postloading to yield VGelA and VGelB NPs. The SEM images of the synthesized NPs revealed a consistent spherical shape for both types of NPs. This spherical shape is preferred since several studies have reported that spherically shaped HA NPs exhibit favorable cytocompatibility and osteogenicity.^33,34^

**Figure 2:**
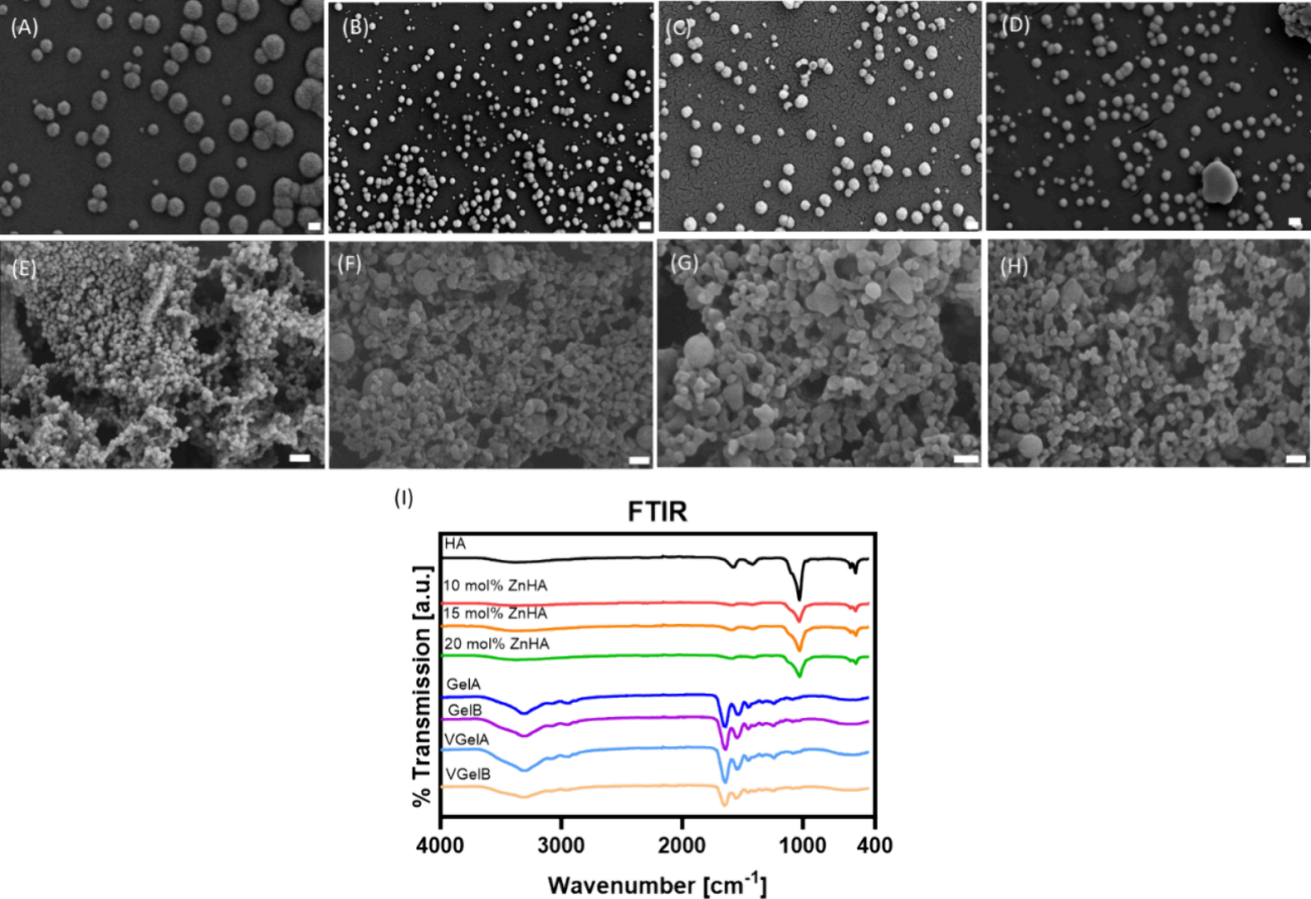
Characteristics of synthesized NP. Scanning electron micrographs displaying the morphology of (A) HA NPs, (B) 10 mol % ZnHA NPs, (C) 15 mol % ZnHA NPs, (D) 20 mol % ZnHA NPs, (E) GelA NPs, (F) GelB NPs, (G) VGelA NPs, and (H) VGelB NPs. Scale bar = 200 nm. Note that all NPs have spherical shapes. (I) FTIR spectra of the synthesized NPs.

The size of all NPs measured from the SEM images was heterogeneous (Figure 2A–F), ranging in dry state from 80 to 250 nm for HA and ZnHA NPs and 75 to 250 nm for Gel NPs. To determine the particle size in the wet state, DLS measurements were employed (Table 1). Both HA and ZnHA NPs exhibited a hydrodynamic size between 200 and 300 nm, which correlates with their particle size in the dry state as determined from the SEM images (Figure 2A–D). However, in the case of Gel NPs, which substantially swell upon contact with water, the DLS measurement indicated sizes of up to 500 nm, which were considerably larger than sizes measured using SEM images (Figure 2E,F). According to Yue et al., particle size crucially determines the process of cellular internalization, and only nanosized particles are able to enter cells via the lysosomal pathways.^35^ Lysosomes can exhibit sizes ranging from 100 to 1500 nm, so a compatible NP size range is required to enable combatting phagolysosomal *S. aureus* bacteria.^36^

**Table 1:** Size and Zeta Potential Measurements of the Synthesized NPs

sample	size (nm)	charge (mV)
HA	204 ± 1	–15 ± 3.4
10 mol % ZnHA	260 ± 16	–10 ± 0.9
15 mol % ZnHA	287 ± 1	–11 ± 0.1
20 mol % ZnHA	265 ± 19	–12 ± 0.2
GelA	449 ± 27	+29 ± 0.3
GelB	360 ± 8	–20 ± 0.4
VGelA	506 ± 6	+27 ± 0.4
VGelB	338 ± 8	–26 ± 0.5

Further insights into the molecular structures of the synthesized NPs were obtained by FTIR (Figure 2G). In general, the broad absorption band between 3000 and 3700 cm^–1^ can be ascribed to O–H stretching. For HA and ZnHA NPs, the sharp peak at 1430 cm^–1^ correspond to CO_3_^2–^ ions which are incorporated in the apatite lattice. The broad band with a shoulder at 950–1100 cm^–1^ is attributed to P–O stretching vibrations of PO_4_^3–^, whereas bending vibrations of PO_4_^3–^ can be observed at the small peaks at around 600 and 555 cm^–1^. These absorptions correspond to phosphate ions in an apatitic lattice. It can be noticed that the OH peaks become less pronounced with increasing zinc content in the ZnHA samples. This observation is attributed to an increasing disorder of the apatitic lattice caused by ionic substitutions.^37^ From these results, it can be concluded that the HA and ZnHA NPs contain a crystalline apatite.^25^ For GelA, GelB, VGelA, and VGelB NPs, an NH-stretching band is observed between 3500 and 3100 cm^–1^. Moreover, two amides are observed with a C=O stretching peak for amide I between 1650 and 1600 cm^–1^ and an N–H deformation peak for amide II at 1550–1500 cm^–1^. The addition of vancomycin did not affect the FTIR spectra.

Furthermore, the zeta potential measurements demonstrated a negative surface charge for HA, ZnHA NPs, and GelB and VGelB NPs, while GelA and VGelA NPs exhibited a positive surface charge (Table 1). HA and ZnHA NPs exhibited negative surface charges, due to the negatively charged carboxyl group derived from the citrate ions adsorbed onto the Ca^2+^ ions from the apatitic NPs.^38^ Meanwhile, the two types of Gel NPs maintained the charges of their gelatin precursors (anionic Gel B and cationic GelA).^39^ Notably, the loading of vancomycin altered the surface charge of VGelA and VGelB to only a minor extent. These opposite charges can be beneficial for future applications of these NPs in self-assembled, mechanical stable networks, also known as colloidal gels.^40^

HPLC results showed that vancomycin loading was more effective onto GelB (31.2 ± 0.06 μg/mL)(vancomycin content:GelB = 3.9 wt %) compared to GelA NPs (9.4 ± 0.07 μg/mL)(vancomycin content:GelA = 1.2 wt %). Vancomycin binds to gelatin by a combination of both electrostatic and hydrophobic interactions.^32^ Apparently, GelB can form more electrostatic bonds with vancomycin due to its anionic nature.

### 3.2 Cytocompatibility of Nanoparticles

**Figure 3:**
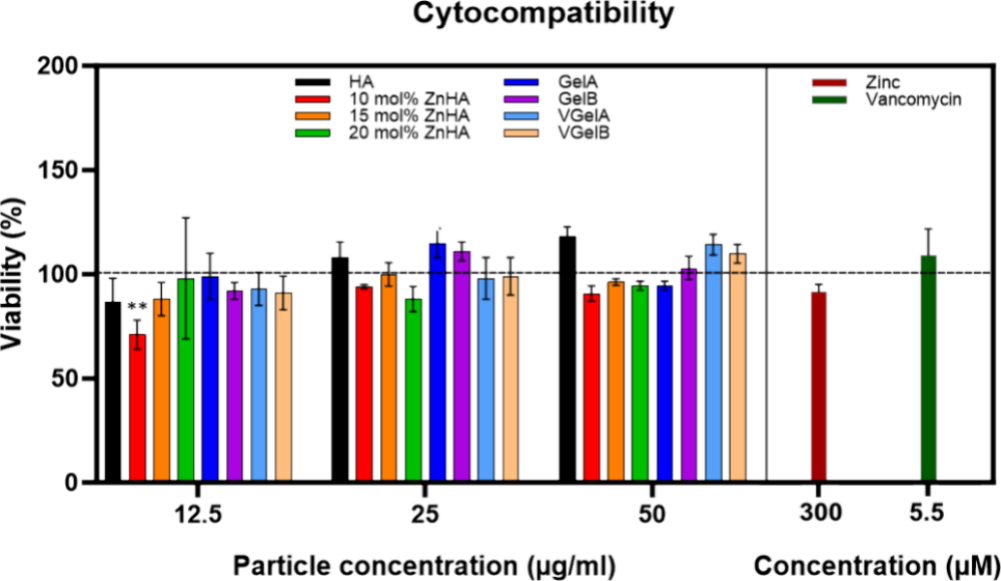
Cytocompatibility of NPs (12.5, 25, and 50 μg/mL) and control zinc (300 μM) and vancomycin (5.5 μM) tested using THP-1 cells.

The cytocompatibility of the synthesized NPs (12.5, 25, 50 μg/mL), zinc (300 μM), and vancomycin (5.5 μM) on THP-1 derived macrophages was evaluated using a CCK8 assay (Figure 3). At similar NP concentrations (12.5, 25, and 50 μg/mL), apart from 10 mol % ZnHA at 12.5 μg/mL, no statistically different (*p* > 0.05) results were observed compared to the untreated control group (100%). Likewise, dissolved zinc and vancomycin controls did not reduce the viability of the cultured cells. These results are in line with previous reports confirming the cytocompatibility of HA,^41^ ZnHA,^25^ gelatin,^42^ zinc,^25^ and vancomycin.^43^ Since the cytocompatibility of the NPs was confirmed for all particle concentrations, subsequent experiments using THP-1 derived macrophages were conducted at the highest particle concentration (50 μg/mL). In addition, we also performed a preliminary pilot test using murine preosteoblast cell line MC3T3-E1 to determine whether the NPs affect the metabolic activity of bone cells (see Supporting Information).

### 3.3 Internalization of Nanoparticles

**Figure 4:**
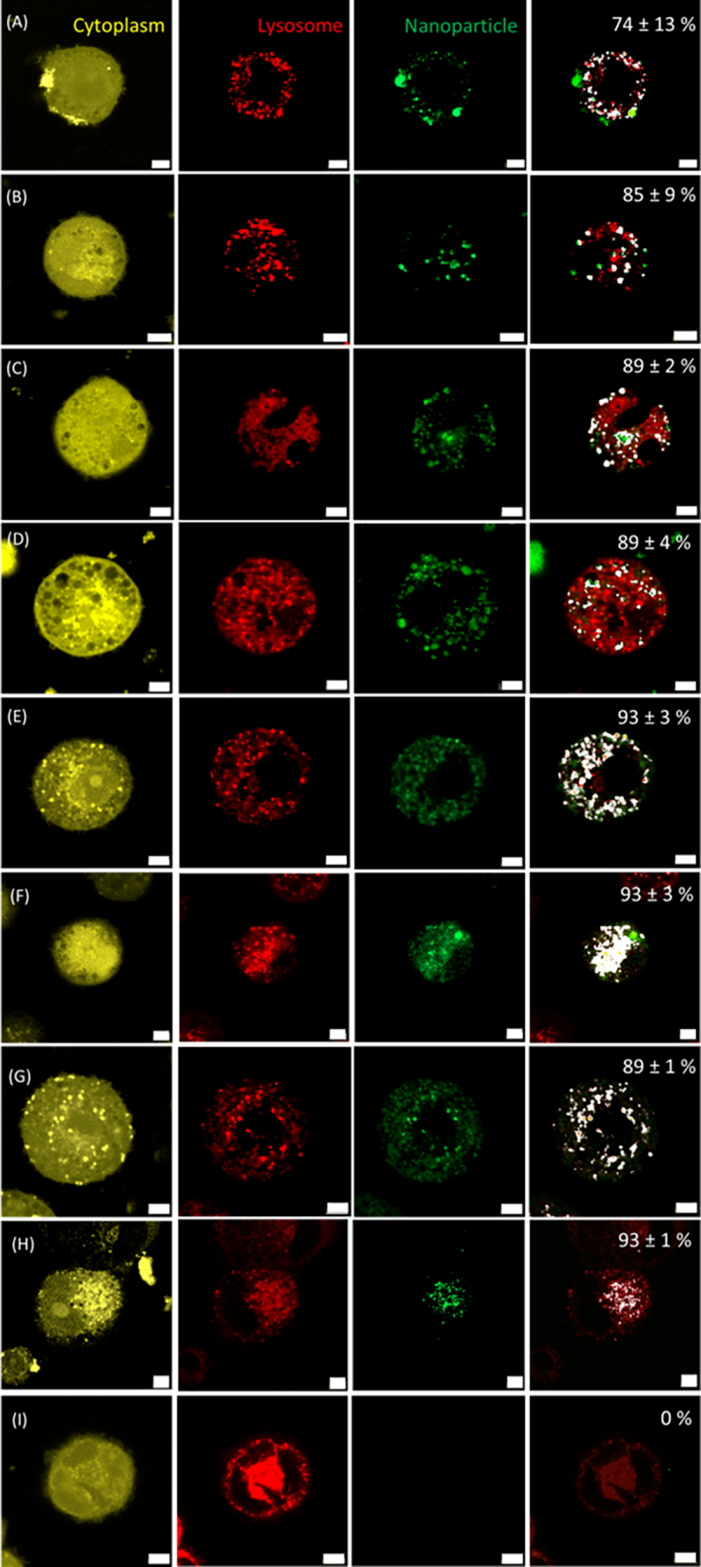
Cellular uptake of various types of NPs by THP-1 cells. (A) HA NPs, (B) 10 mol % ZnHA NPs, (C) 15 mol % ZnHA NPs, (D) 20 mol % ZnHA NPs, (E) GelA NPs, (F) GelB NPs, (G) FITC-VGelA NPs, (H) FITC-VGelB NPs, and (I) FITC-vancomycin. The cytoplasm is visualized in yellow, lysosomes in red, and NPs in green. An overlay of the lysosomal signal and NP signal was depicted in white and quantified. All NPs displayed high internalization as displayed in percentages at the top right of each experimental group. Note that all NPs were abundantly internalized by THP-1 cells and colocalized with the lysosome. Vancomycin was not detected intracellularly without a carrier. Scale bar is 5 μm.

All NPs did not show any toxic effects on the THP-1 cells. At a similar concentration of 50 μg/mL, the internalization and intracellular localization of the synthesized NPs within THP-1 derived macrophages was evaluated using confocal microscopy of the cell cytoplasm, lysosomes, and fluorescently labeled NPs. Overlay images were produced, combining the separate signals from the lysosomes and NPs, and the amount of colocalization was subsequently quantified (Figure 4). All results revealed a high degree of colocalization of lysosomes and NPs, while we observed a significantly lower cellular uptake of HA NPs (∼74%) compared to GelA, GelB, and VGelB NPs, which were internalized for ∼90% by the cells (*p* < 0.05). Noteworthy, no signal of dissolved FITC-vancomycin was observed in THP-1 cells, confirming that this antibiotic is hardly internalized by these cells. Vancomycin could only be detected within cells by utilizing an NP carrier such as GelA or GelB NPs.

These results are consistent with the literature describing lysosomal uptake of HA NPs after endocytosis.^44^ The intracellular uptake of Gel NPs has also been previously described in mouse preosteoblastic cells^45^ and THP-1 derived fused macrophages,^46^ and similarly occurs through lysosomal uptake. The lysosomal environment should stimulate the release of the bactericidal agents. In previous work of our group, it was shown that HA NPs rapidly dissolve at low pH resulting in abundant release of zinc ions, which does not occur at a physiological pH of 7.4.^25^ Additionally, it was shown in previous work that vancomycin releases at a faster rate from Gel NPs at acidic vs alkaline conditions.^32^

Remarkably, we did not observe any statistically significant difference (*p* > 0.05) regarding the uptake of positively or negatively charged NPs. For gelatin, this observation is consistent with previous studies reporting that the surface charge of Gel NP surface charge does not affect the efficacy of cellular uptake.^45^ Moreover, in our study, we immersed NPs in culture media, potentially introducing the formation of a protein corona layer around the NP, thus neutralizing its surface charge.^47^

### 3.4 Coculture Model of THP-1 Derived Macrophages Containing Phagocytosed *S. aureus*

#### 3.4.1 Validation of the Coculture Model

A model of intracellular *S. aureus* bacteria phagocytosed by human THP-1 derived macrophages was adapted from the previously reported literature. These studies predominantly employed a ratio of bacteria to THP-1 cells of 4:1,^4,30^ whereas we used a lower ratio of 1:1 to avoid THP-1 cell death due to excessive bacterial uptake (results not shown).

**Figure 5:**
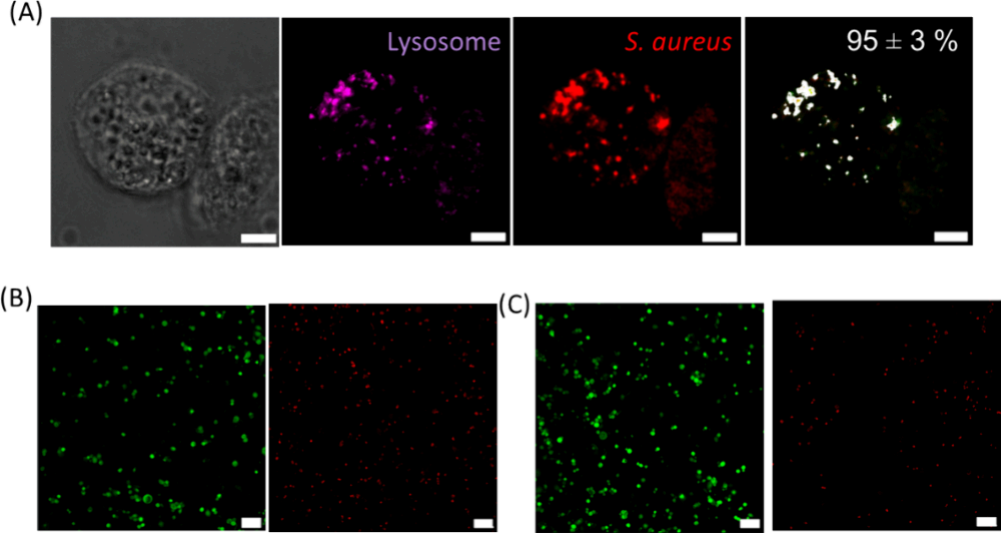
(A) Intracellular localization of *S. aureus*-mCherry in THP-1 cells. Cells were visualized by bright-field microscopy, lysosomes were visualized in magenta, and *S. aureus*-mCherry in red. An overlay of lysosomal signal and bacteria signal was depicted in white, quantified, and displayed on the top right of the image. Note the pronounced colocalization of *S. aureus*-mCherry and the lysosomes. Scale bar corresponds to 5 μm. (B) LIVE/DEAD staining of THP-1 cells and (C) THP-1 cells with phagocytosed *S. aureus*-mCherry bacteria. Viable cells are shown in green, and dead cells are in red. Note that the viability of THP-1 cells with and without phagocytosed *S. aureus*-mCherry was similar. Scale bar corresponds to 100 μm.

To unravel the intracellular localization of *S. aureus* after phagocytosis, we employed confocal microscopy. To facilitate subsequent imaging, a strain of mCherry-labeled *S. aureus* bacteria was used. To investigate the extent of phagocytosis in this model, cells were visualized using bright-field microscopy, while lysosomes and *S. aureus*-mCherry were visualized using confocal fluorescence microscopy. In overlay images, the amount of colocalization of lysosomes and *S. aureus*-mCherry bacteria was quantified as 95 ± 3%, indicating a highly efficient internalization of bacteria ending up inside lysosomes (Figure 5A), confirming the literature identifying *S. aureus* as phagolysosomal bacteria.^36^ To assess the viability of THP-1 cells following phagocytosis, LIVE/DEAD staining was conducted. A comparative analysis between bacteria-free THP-1 cells (Figure 5B) and THP-1 cells containing internalized *S. aureus*-mCherry (Figure 5C) did not reveal any observable difference regarding cell viability. Consequently, we concluded that the phagocytic process did not cause toxicity, thereby affirming the suitability of this model for further investigations.

#### 3.4.2 Visualization of Intracellular *S. aureus*-mCherry and Nanoparticles

**Figure 6:**
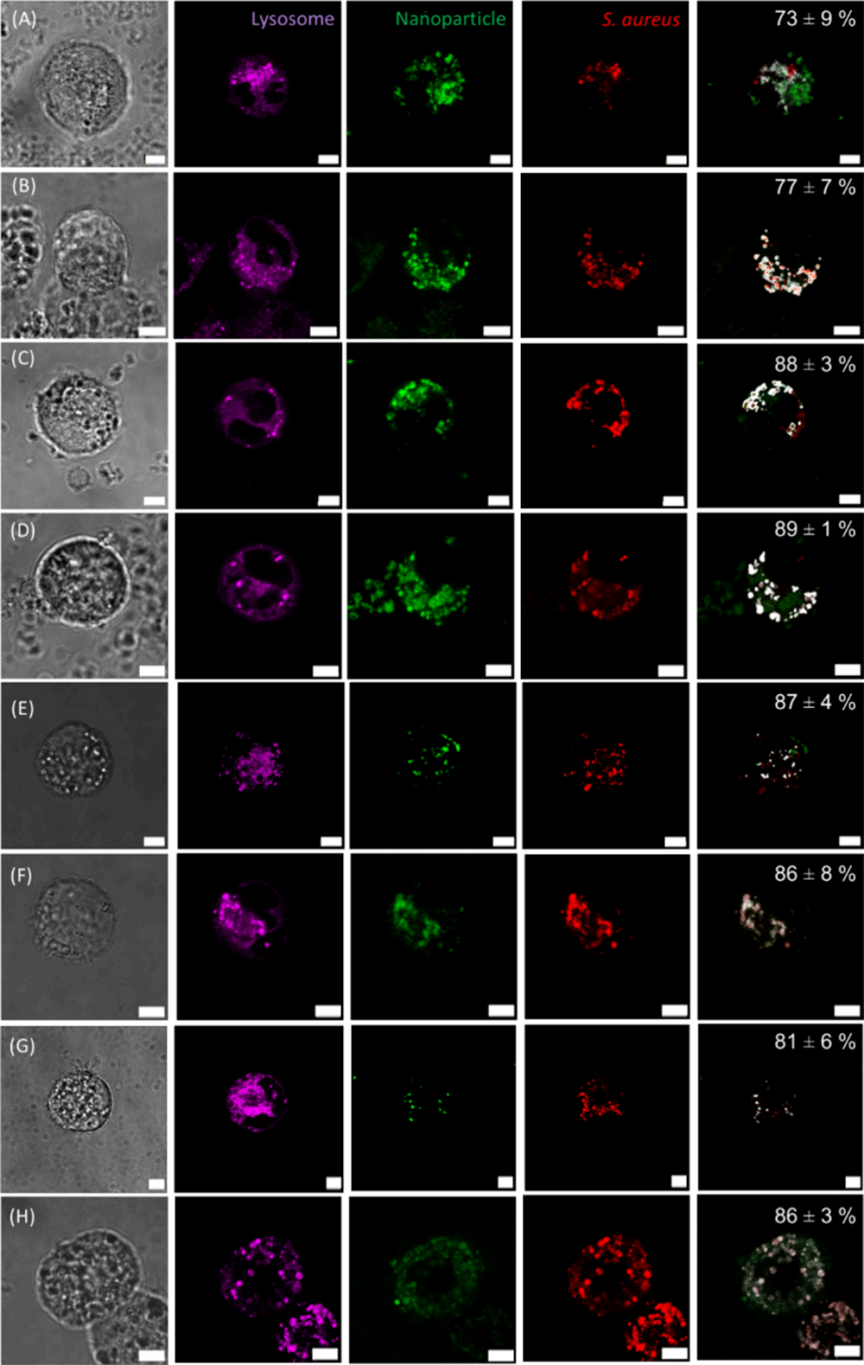
THP-1 cells with phagocytosed *S. aureus*-mCherry cultured with (A) HA NPs, (B) 10 mol % ZnHA NPs, (C) 15 mol % ZnHA NPs, (D) 20 mol % ZnHA NPs, (E) GelA NPs, (F) GelB NPs, (G) FITC-VGelA NPs, and (H) FITC-VGelB NPs. Cells were visualized using bright-field microscopy, lysosomes were depicted in magenta, synthesized NPs and antibacterial agents were in green, and *S. aureus*-mCherry was in red. An overlay of the NPs signal with the *S. aureus*-mCherry signal was depicted in white and quantified. All NPs were internalized at high efficiency, as displayed in percentages at the top right of each experimental group. Colocalization of the various NPs was similar. Scale bar corresponds to 5 μm.

Following the successful phagocytosis of *S. aureus*-mCherry, the synthesized NPs were supplemented with the culture media to facilitate their internalization by human THP-1 derived macrophages. Confocal microscopy was employed to observe their intracellular localization (Figure 6), and as before, the THP-1 cells were visualized using bright-field microscopy, while lysosomes, NPs, and *S. aureus*-mCherry were observed in confocal fluorescence microscopy. It should be stressed that *S. aureus* bacteria express the mCherry protein, which renders them visible using confocal microscopy. When the bacteria die, the mCherry protein will be no longer detected.^48,49^ An overlay analysis of NPs and *S. aureus*-mCherry was conducted and quantified. Notably, all NPs demonstrated high degrees of colocalization with *S. aureus*-mCherry, confirming their colocalization within the lysosomes of the THP-1 cells. We did not observe statistically significant differences between the various types of NPs (*p* > 0.05). Yao et al. investigated the phagocytic capacity of THP-1 cells subsequent to NP uptake, revealing a significant reduction in bacterial phagocytosis by THP-1 cells post-NP uptake.^50^ Although this study performed a different phagocytosis protocol, the strong fluorescent signal of NPs within the THP-1 cells underscored that the initial phagocytosis of *S. aureus* did not impede subsequent uptake of the synthesized NPs, which is crucial for the envisaged clinical application.

#### 3.4.3 Intracellular Antibacterial Activity of Nanoparticles

In order to investigate the intracellular bactericidal efficacy, infected cells were treated with various types of NPs. To assess the extent of killing of intracellular bacteria, cellular lysis was performed, and the intracellular *S. aureus*-mCherry bacteria were spot-plated to quantify the number of CFU. First, we confirmed that the NPs were cytocompatible with the THP-1 cells to ensure that subsequent reduction of intracellular bacteria, as assessed by plating and CFU counting, can fully be attributed to intracellular bacterial eradication rather than cellular toxicity. Moreover, gentamycin was added to the culture media to eradicate extracellular bacteria. Gentamycin is unable to cross the cell membrane, which impedes the interaction between intracellular bacteria and gentamycin.^51,52^ Spot-plating of the culture media surrounding the cells confirmed the absence of extracellular bacteria in all groups after 24 h of culture (results not shown). This control experiment is crucial to confirm that all assessment of plated bacteria corresponds to intracellular bacteria instead of confounding circumstances such as extracellular bacteria adhering to the external surface of the cells. Although the introduction of gentamycin could have led to interactions of gentamycin with the NPs, such an effect seems unlikely based on previous literature which did not report any effects of gentamycin on zinc^53^ or vancomycin.^54,55^

**Figure 7:**
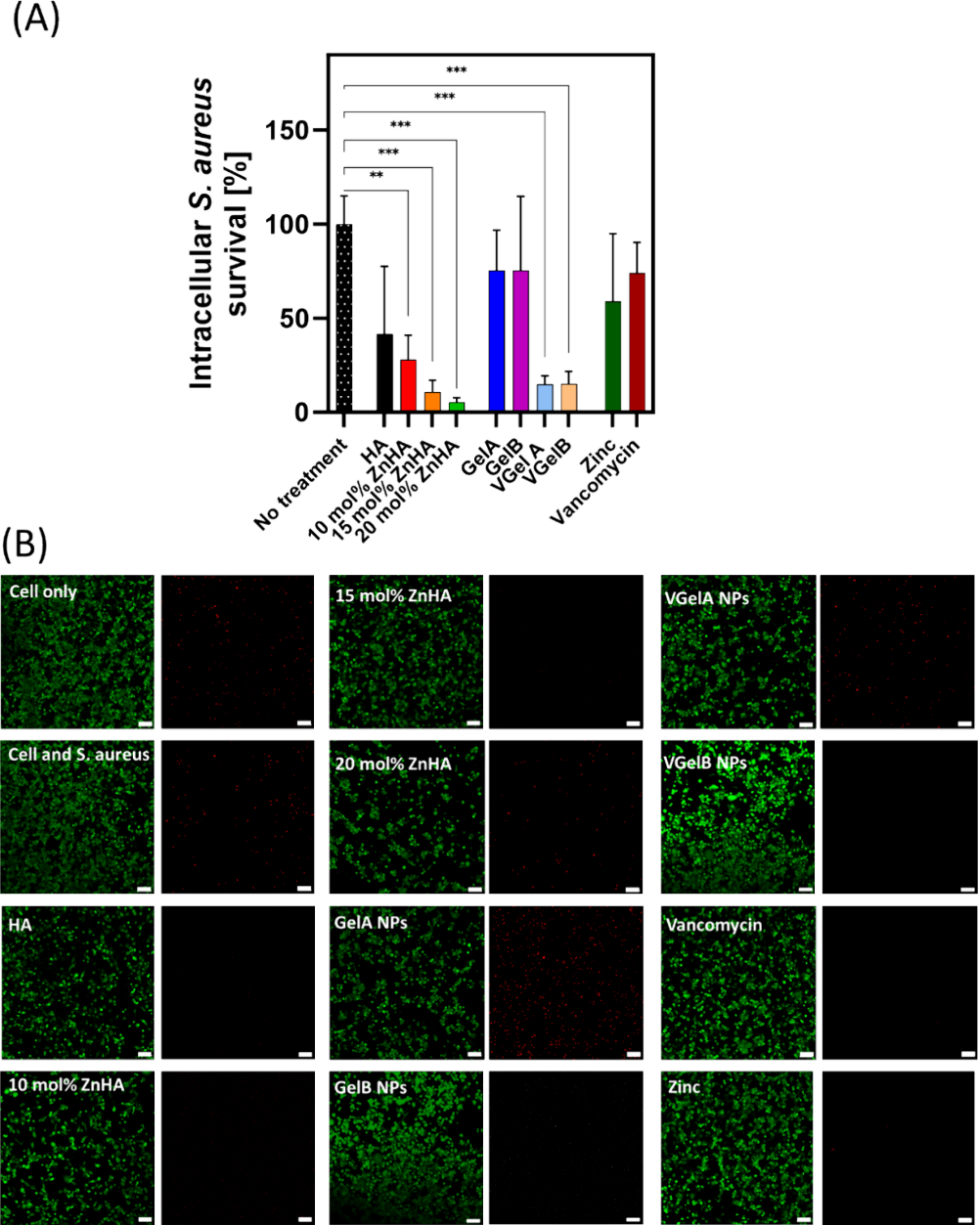
(A) Intracellular survival of *S. aureus*-mCherry 24 h after the addition of various types of NPs. Note that NPs containing vancomycin or zinc exhibit strong bactericidal effects, whereas direct addition of dissolved vancomycin or zinc did not. (B) LIVE/DEAD staining showing alive (green) and dead (red) THP-1 cells containing phagocytosed *S. aureus*-mCherry cultured in the presence of NPs or dissolved zinc or vancomycin for 24 h. Viability of the THP-1 cells was not affected by either of the NPs. Scale bar corresponds to 100 μm.

Figure 7 shows the impact of the synthesized NPs on the survival of intracellular *S. aureus*-mCherry. Zinc-free HA NPs and ZnHA NPs with different zinc concentrations (10, 15, and 20 mol %) were added to the coculture to investigate the intracellular bactericidal effect of zinc. All zinc-doped NPs substantially (*p* < 0.01) reduce the intracellular survival of *S. aureus*-mCherry. Among these experimental groups, the group receiving 20 mol % ZnHA NPs displayed the lowest CFU count, which confirms the dose-dependent intracellular bactericidal effect of zinc ions. HA NPs did not reduce the number of viable *S. aureus* bacteria in a statistically significant manner.

Vancomycin-free Gel NPs did not reduce the intracellular bacterial survival as compared to the untreated control group. However, vancomycin-loaded VGelA and VGelB NPs significantly reduced the intracellular viability of bacteria as compared to the untreated control group (*p* < 0.01), confirming the bactericidal effect of vancomycin. Previous literature described other carriers that can successfully transport vancomycin into the cell, such as cell-penetrating polypeptides,^56^ liposomes,^57^ inorganic nanoparticles,^58^ metal nanoparticles,^59^ and dendrimers.^60^ However, we deliberately selected bone-like nanoparticles derived from organic and inorganic constituents of bone, i.e., hydroxyapatite and gelatin nanoparticles. Noticeably, the intracellular killing by VGelA and VGelB NPs was comparable, even though VGelB NPs were more efficiently loaded with vancomycin.

While *S. aureus* is typically considered a phagolysosomal bacterium, it may also reside in the cytoplasm. Our NPs exhibit pH-responsive behavior where low pH values trigger the release of antibacterial agents, suggesting that the primary antibacterial effect occurs within the lysosome. However, NPs can also generate reactive oxygen species or interact with bacterial cell walls due to their surface charge.^61,62^ This suggests that our NPs may exhibit antibacterial activity not only within lysosomes but also in the cytoplasm.

Finally, it should be emphasized that the control group, entailing dissolved zinc and vancomycin, did not significantly reduce the survival of intracellular *S. aureus*-mCherry, even though the total amount of systemically derived zinc and vancomycin was considerably higher in these controls as compared to the groups treated with NPs. Notably, all types of NPs were cytocompatible with THP-1 cells (Figure 7B).

For future research, the noninvasive application of these two NPs is essential to allow for future clinical translation. To this end, the therapeutic efficacy of colloidal gels composed of these two NPs to cure bone infection should be tested in suitable animal models for bone infection, which can be established, for instance, by injection of intracellular *S. aureus*.^63^

## 4 Conclusions

In this study, two distinct nanoparticles (i.e., zinc-doped hydroxyapatite and vancomycin-loaded gelatin nanoparticles) were employed to treat intracellular infection in a thoroughly validated coculture model of human THP-1 derived macrophages infected with *S. aureus* bacteria. Both types of particles eradicated intracellular *S. aureus* bacteria in a highly effective manner. These results evidenced that both hydroxyapatite and gelatin NPs are suitable carriers for antibacterial ions and antibiotics, respectively, to facilitate the effective treatment of intracellular bone infections. These findings open up new avenues of research on novel treatments of chronic and recurrent intracellular bone infections in human patients. Future research should determine intracellular zinc and vancomycin release kinetics from the NPs, in order to further enhance the therapeutic efficacy by achieving synergistic effects.

## Supporting Information Available

Additional preosteoblast MC3T3 cell experiment details, materials and method, and results (PDF)

## Abbreviations

NPs: nanoparticles
Gel: gelatin
HA: hydroxyapatite
ZnHA: zinc-doped hydroxyapatite
VGel: vancomycin-loaded gelatin
GelA: gelatin type A
GelB: gelatin type B
EDC: 1-ethyl-3-(3-dimethylaminopropyl)carbodiimide
NHS: 1 mM *N*-hydroxysuccinimide
PBS: phosphate buffered saline
SEM: scanning electron microscopy
DLS: dynamic light scatter
FTIR: Fourier transmission infrared
HPLC: high pressure liquid chromatography
RPMI: Roswell Park Memorial Institute
FBS: fetal bovine serum
PMA: phorbol 12-myristate 13-acetate
FITC: fluorescein 5(6)-isothiocyanate
TSB: trypsin soy broth
CFU: colony forming units
OD620: optical density at 620 nm
