# Supplementary material for: Antibacterial Activity of Zinc-Doped Hydroxyapatite and Vancomycin-Loaded Gelatin Nanoparticles against Intracellular Staphylococcus aureus in Human THP-1 Derived Macrophages
Source: ACS Appl Nano Mater. 2024 Sep 11;7(18):21964–74. doi: 10.1021/acsanm.4c03941 (PMC11443495; doi:10.1021/acsanm.4c03941)
Supplement: Supplementary file 1 — an4c03941_si_001.pdf [file an4c03941_si_001.pdf]

Supporting Information

Antibacterial Activity of Zinc-Doped Hydroxyapatite and Vancomycin-Loaded Gelatin Nanoparticles Against Intracellular Staphylococcus aureus in Human THP-1 Derived Macrophages

*Lizzy A.B. Cuypers [a], Leonie de Boer [b], Rong Wang [a], X. Frank Walboomers [a], Fang Yang [a], Sebastian A.J. Zaat [b], Sander C.G. Leeuwenburgh [a]**

[a] Radboud university medical center, Research Institute Medical Innovations, Department of Dentistry-Regenerative Biomaterials, Philips van Leydenlaan 25, 6525 EX Nijmegen, The Netherlands

[b] Department of Medical Microbiology and Infection Prevention, Amsterdam Institute for Immunology and Infectious Diseases, Amsterdam University Medical Center, University of Amsterdam, Meibergdreef 9, 1105 AZ Amsterdam, The Netherlands

* Corresponding author: sander.leeuwenburgh@radboudumc.nl

Table of Contents

A. Materials and Methods……………………………………………………..……………S2

B. Results………………………………………………………………………………S2

A. Materials and Methods:

Murine pre-osteoblast cell line MC3T3-E1 subclone 4 (CRL-2593, American Type Culture Collection (ATCC), Manassas, VA, USA) was maintained in complete α-MEM supplemented with 10% FBS. The medium was replaced every 3-4 days and the cultures were incubated at 37˚C in a humidified atmosphere containing 5% CO_2. The cells were passaged by trypsinization when reaching a confluence of 80%.

After trypsinization, the cells were seeded in a 96 well plate at a concentration of 10.000 cells/cm^2 and incubated overnight to ensure cell attachment. After overnight culture, media were replaced by media containing NPs at concentrations of 12.5, 25 and 50 μg/ml. The cytocompatibility of MC3T3 cells exposed to NPs (no treatment group as control) was assessed using a cell counting kit assay (CCK8; 150 NaCl, 5 mM WST-8 and 0.2 mM 1-methoxy PMS in deionized water) at day 1 and 5. On the pre-determined time points, the media were replaced by fresh media supplemented with 10% CCK8 reagent and incubated for 2h. The supernatants were collected, and the absorbance was measured at a wavelength of 450-490 nm using a spectrophotometer (BioTek, Winooski, VT, USA).

B. Results:

Figure S1 shows that all NPs were cytocompatible with MC3T3-E1 pre-osteoblast cells. Interestingly, all HA-based NPs (i.e. HA and ZnHA NPs) even displayed enhanced metabolic activity at day 5 as compared to day 1.

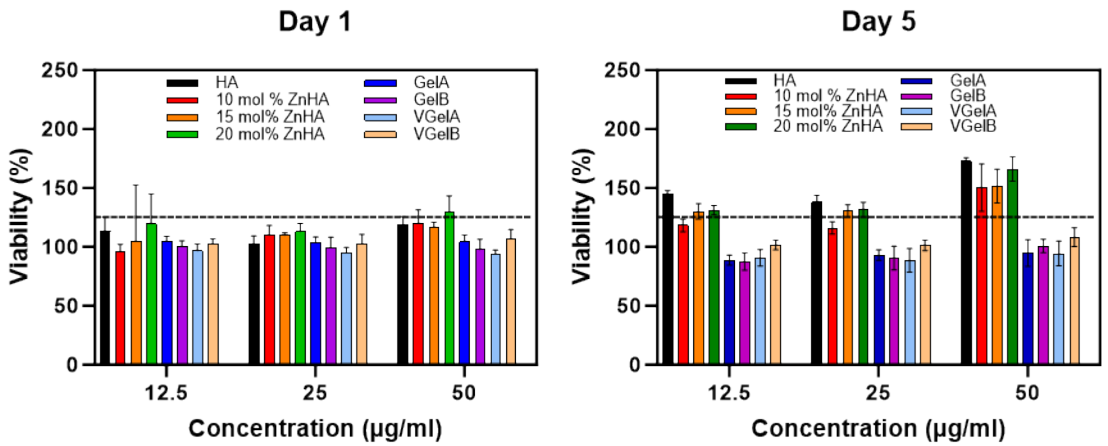

Figure S1: Cytocompatibility of NPs (12.5, 25 and 50 μg/ml) tested using MC3T3 pre-osteoblasts.
